# Switchable directional scattering of electromagnetic radiation with subwavelength asymmetric silicon dimers

**DOI:** 10.1038/srep18322

**Published:** 2015-12-10

**Authors:** Pablo Albella, Toshihiko Shibanuma, Stefan A. Maier

**Affiliations:** 1 The Blackett Laboratory, Imperial College London, London SW7 2AZ, UK

## Abstract

High refractive index dielectric nanoparticles show high promise as a complementary nanophotonics platform due to compared with plasmonic nanostructures low absorption losses and the co-existence of magnetic and electric resonances. Here we explore their use as resonantly enhanced directional scatterers. We theoretically demonstrate that an asymmetric dimer of silicon nanoparticles shows tuneable directional scattering depending on the frequency of excitation. This is due to the interference between electric and magnetic dipoles excited in each nanoparticle, enabling directional control of the scattered light. Interestingly, this control can be achieved regardless of the polarization direction with respect to the dimer axis; however, difference in the polarization can shift the wavelengths at which the directional scattering is achieved. We also explore the application of such an asymmetric nanoantenna as a tuneable routing element in a nanometer scale, suggesting applications in optical nanocircuitry.

Recent developments in nanophotonics and plasmonics^1^ have provided versatile tools to manipulate and control light at the nanoscale^2^. The collective oscillations of conduction electrons at the surface of metallic nanoparticles, so-called localized surface plasmon resonances, can create an intense electric field in small areas beyond the diffraction limit of light and significantly increase the particle’s extinction cross section. Plasmonic resonances are hence capable of improving the efficiency of solar cells, enhancing the sensitivity of spectroscopic methods such as surface enhanced Raman scattering and fluorescence, and also form one of the underlying building blocks for nanometer scale optical circuitry^3^,^4^,^5^. Plasmonic nanoparticles have also been employed to control the direction of scattered light. One well known example is optical analogues to Yagi-Uda antennas^6^,^7^. More compact structures such as bimetallic dimer and V-shaped metallic nanoantennas also show unidirectional scattering due to the phase difference of two multipolar resonances excited in the particles^8^,^9^. However, energy losses specifically in the visible and NIR regimes are unavoidable because of free electron absorption in the metal^10^.

Very recently, particles of high refractive index dielectric materials have been studied for nanophotonics applications as a complementary platform to plasmonic materials^11^,^12^,^13^,^14^,^15^,^16^,^17^,^18^. In addition to the several practical benefits, such as the capability of using mature semiconductor fabrication techniques and the cheap cost of the material, dielectrics possess a variety of advantages when compared to metals. Resonances in high refractive index dielectrics are excited with low energy losses even in the optical and NIR regions, owing to the absence of free electron absorption. Furthermore, magnetic resonances can be excited, originating from the rotation of the displacement current^19^,^20^. These magnetic resonances are described well by the Mie theory. Even a simple spherical, cylindrical or cubic structure of a dielectric material with refractive index larger than two, can show magnetic resonant modes^21^, whereas metallic nanoparticles require complicated structures (e.g. split ring resonators) to show magnetic resonances. Due to the existence of the magnetic resonances and their interference with the electric ones, single Si or GaAs spherical particles have shown the capability of controlling the scattering ratio between forward and backward direction^22^,^23^,^24^,^25^,^26^. Moreover, dimer structures of high refractive index dielectric nanoparticles sustain strong interaction between the resonances excited in each constituent particle, leading to intense electric or magnetic fields at the gap, similar to plasmonic hot spots, but without significant heating of the structure. Such dimer assemblies have been applied to sensitive spectroscopic applications or the generation of Fano resonances^27^,^28^,^29^,^30^,^31^. However, directional control of the scattered field away from the incident direction has not been demonstrated with simple dielectric nanostructures. The rotation of the scattering direction could be favourable for highly sensitive sensing schemes and nanoantennas in optical nanocircuits.

In this paper, we reveal that a dielectric nanodimer consisting of nanoparticles with different dimensions can scatter light directionally to either the right or left direction by tuning the incident wavelength. The underlying reason is that amplitude and phase differences of electric and magnetic resonances in dielectric nanoparticles can be tuned by changing geometrical dimensions. By carefully designing the dimer configuration, therefore, the direction of the scattered light becomes tuneable due to the interference between the dipoles excited in each particle. Here, we carry out full theoretical calculations using an analytical dipole-dipole model to determine a favourable configuration for highly directional scattering tuneable via the incident wavelength. We also propose a practical configuration of asymmetric dimers as a nanoscale routing element for electromagnetic radiation. Directional control of the scattered light combined with low energy losses presented in this paper can facilitate the development of efficient sensors, waveguides and optical circuits at the nanometer scale.

## Results

The polarizability of dielectric nanoparticles strongly depends on their dimensions^32^. Therefore, phase differences in the polarization should appear when two dielectric particles of different dimensions are placed in proximity. Furthermore, electric and magnetic dipoles in dielectric nanoparticles are excited at different wavelengths and perpendicular to each other. Hence, a change in incident polarization could lead to a shift of the wavelength at which the directional scattering is achieved.

**Figure 1:**
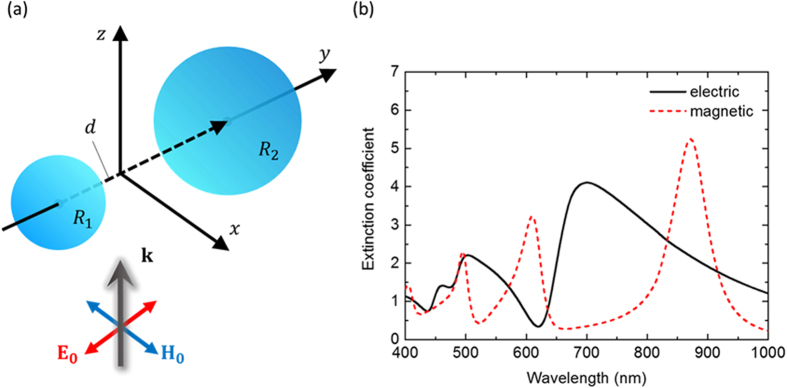
(**a**) A schematic image of a dimer of silicon spherical nanoparticles and the propagation direction of incident radiation (p-polarized). (**b**) Extinction spectra of electric and magnetic dipoles calculated using an analytical dipole-dipole model.

First, we theoretically investigated a dimer system consisting of spherical Si nanoparticles, based on an analytical dipole-dipole model that we developed in a previous work^27^. Here the electromagnetic responses of silicon spherical nanoparticles were substituted by electric and magnetic dipoles, justified by the large ratio of wavelength to particle dimension leading to less significance of higher order modes. The diameters and gap separation of the dimeric structure are parameters that can affect the resonant characteristics of the dielectric dimer and, hence, the properties of light steering (see Figure S1 and S2 in Supplementary Information). In our optimised exemplary system, two nanospheres of radius *R*_1_ and *R*_2_ (*R*_1_ < *R*_2_) are placed at **r**_1_ and **r**_2_ in a Cartesian coordinate space. The configuration used in the theoretical analysis is described in Fig. 1a. The radii of the smaller and larger spheres are *R*_1_ = 75 nm and *R*_2_ = 115 nm, respectively, and these two spheres are separated by a gap of *d* = 8 nm in air. Fig. 1b shows the calculated extinction spectra of electric and magnetic dipoles excited in a dimer of silicon spherical nanoparticles. The two spectra show three peaks; the resonances at the shortest and longest wavelengths are attributed to the excited dipoles in the larger particle, and the resonances at intermediate wavelengths correspond to the dipoles excited in the smaller particle. The resonant wavelength of the dielectric particle strongly depends on their dimensions since the oscillation or rotation of displacement current inside the particle results in the excitation of electric or magnetic resonances^19^,^20^. Note that the intensities of these resonances are comparable between the two spheres and, hence, suitable for generating constructive or destructive interferences.

**Figure 2:**
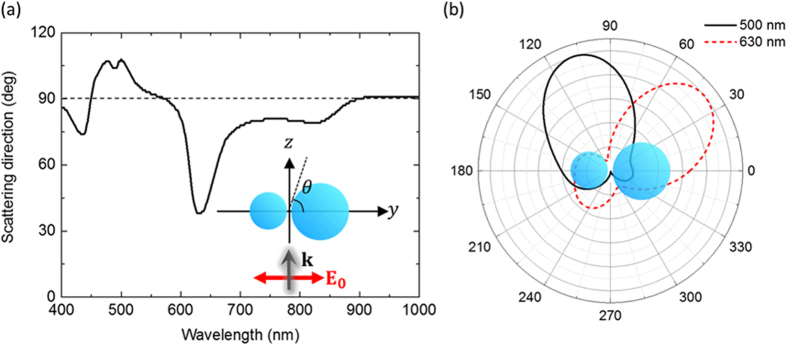
(**a**) Maximum angle of scattered intensity on the y-z plane projection. (**b**) Far field radiation patterns of the scattered light at λ = 500 nm and λ = 630 nm.

In Fig. 2a, we plot the angle of maximum scattering intensity in the projection of the far field pattern on the y-z plane. Here the scattering angle of 90° corresponds to the forward scattering, and the two quadrants (0°–90° and 90°–180°) correspond to the scattering hemisphere of the larger and smaller particles, respectively. The direction of the scattered light bends left (108°) or right (38°) at λ ~ 500 nm and λ ~ 630 nm, respectively. Far field patterns on the y-z plane at these wavelengths are shown in Fig. 2b. At λ ~ 500 nm, the scattered light was directed to the smaller sphere but suppressed in the direction of the larger sphere. Meanwhile, a completely opposite result was achieved at λ ~ 630 nm; the light scattered to the smaller sphere was suppressed but the light to the larger sphere was intensified. This means that the direction of the scattered field can be tuned by changing incident wavelength.

**Figure 3:**
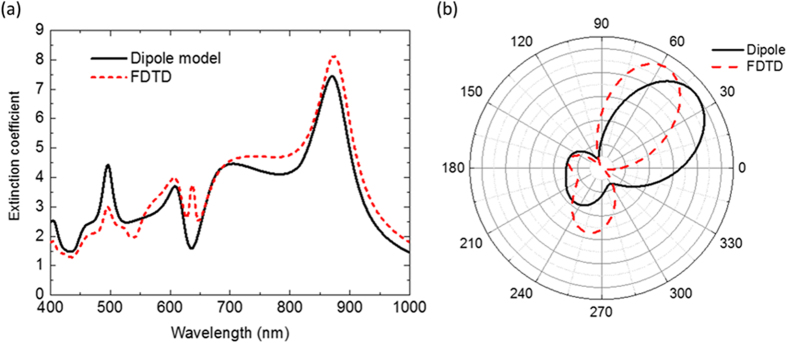
Comparison of the (a) extinction spectrum and (b) far field radiation pattern of the scattered field at 630 nm, calculated by the analytical dipole-dipole model (black solid line) and by the full numerical FDTD method (red dash line).

In order to show the validity of the dipole coupling model to understand the physical origin of the light directionality we compare full finite-difference time-domain method (FDTD) calculations with the dipole-dipole interaction model. In Fig. 3a we plot the extinction and the radiation patterns calculated with both methods. These spectra basically shows a good agreement. A distinct peak at λ = 640 nm is observed only in the FDTD simulation, which corresponds to the magnetic quadrupolar resonance of the larger sphere^27^. In Fig. 3b we perform a similar comparison but for the radiation patterns at λ = 630 nm. In both cases we see a clear steering of the light. Notice that the dipolar model clearly overestimates the rotation angle due to the absence of the magnetic quadrupolar mode that may interfere in a destructive way at this wavelength (see Fig.S3 of supplementary information). However, as we will show later, in a real situation of a dimer of silicon disks on a substrate, the steering of light is of around 30°. This is enough to excite a certain position selectively, showing that the influence of higher order modes does not change our conclusion.

To understand the basic mechanism of this directional scattering, the phase difference of the excited dipoles was analysed. The electric dipoles excited along to the dimer axis were found not to have major contributions to the scattered light along the y-axis direction. In a similar manner, the electric dipoles excited along the z-axis by the magnetic dipoles can be neglected since their intensity in the analysed illumination configuration is very small. Therefore, we focus only on the magnetic dipoles excited along the x-axis. The oscillation of the magnetic dipole first generates magnetic field as a function of time and space, which in turn induces electric field. Since the generated electromagnetic field propagates to the far field, the oscillation of the magnetic dipole can contribute to the scattered field intensity. It should be noted that excitation of magnetic resonances is possible due to the high refractive index of the dielectric material, whereas in the case of plasmonic nanostructures this is not possible because the field does not penetrate enough as to generate sufficient displacement currents for the excitation of magnetic resonances^22^.

**Figure 4:**
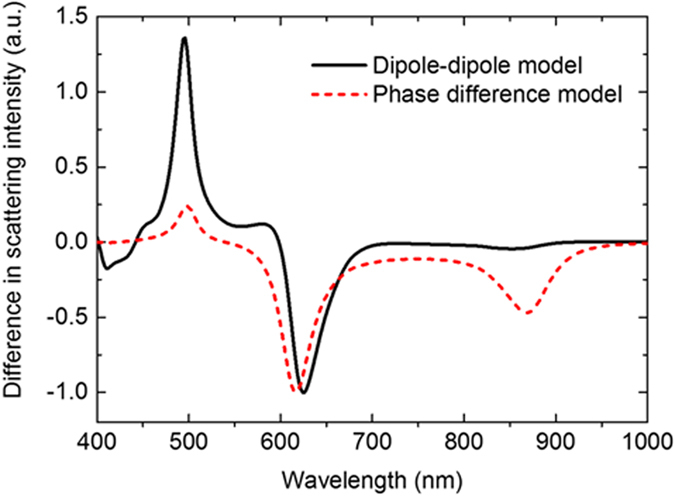
Comparison of the difference in scattering intensity calculated by the analytical dipole-dipole model (black solid line) and the phase difference model (red dash line).

The phase difference generated by each particle, ΔΦ_1_ and ΔΦ_2_, can be calculated from the complex value of the magnetic polarizabilities. Interference term was attributed to *ΔΦ  ±  kd*, where *ΔΦ*  *=* *ΔΦ*_*1*_*–ΔΦ*_*2*_ and *k* is the wavenumber of the incident light. Directional scattering is achieved if constructive interference occurs in one direction and destructive interference in the other. Figure 4 shows a comparison of the scattered light intensity propagating towards 180° and the one towards 0°, given by the far field calculated with the analytical dipole-dipole model and with the approximated phase difference model. These two plots qualitatively agree well specifically in terms of the wavelengths at which directional scattering occurs. The small discrepancy between the two plots can be attributed to not considering the presence of electric dipoles and their very small interaction with magnetic dipoles.

**Figure 5:**
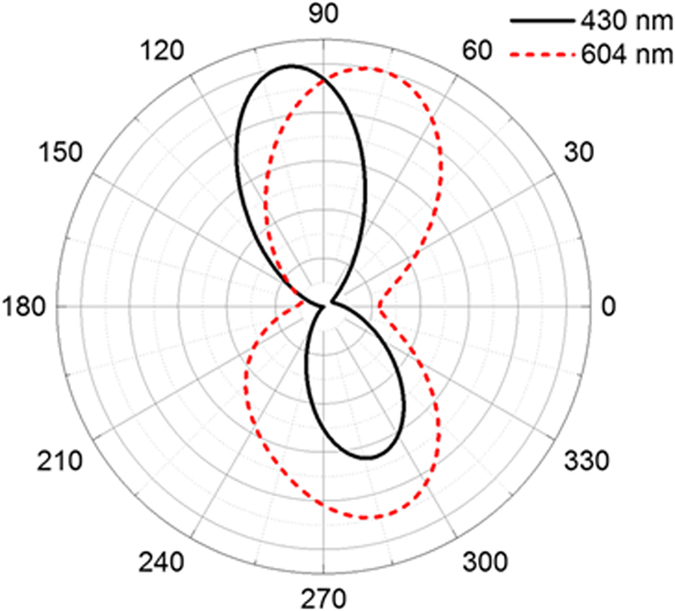
Far field radiation patterns of the scattered light at λ = 430 nm and λ = 604 nm excited with s-polarization.

Different polarization configurations of the exciting radiation were also investigated. In this case, the dimer structure was the same as the previous one, but the incident light is configured with electric polarization perpendicular to the dimer axis (s-polarized). Figure 5 shows the far field distribution of the scattered light which was projected on the y-z plane at 430 nm and 604 nm. The scattered light was also directed to the larger (by ~13°) or smaller (~10°) sphere, depending on the incident wavelength. In this polarization configuration, the directional scattering could be mainly attributed with the interference between the two electric dipoles excited perpendicular to the dimer axis. Since the electric dipoles resonate at shorter wavelengths than the magnetic dipoles, the wavelengths at which the directional scattering was achieved were blue-shifted. In addition, the degrees of the rotation were slightly smaller. The obtained results, therefore, suggest that incident polarization provides, in addition to excitation wavelength, another route for tuning the spectral response of directional scattering.

## Discussion

In an optical circuit, a switchable nanoantenna could be responsible to guide the light to an appropriate direction. In integrated optics, light is usually guided by silicon waveguides. In this section, we investigate the possibility to use our asymmetric dielectric dimer as a switchable nanoantenna for an optical circuit.

**Figure 6:**
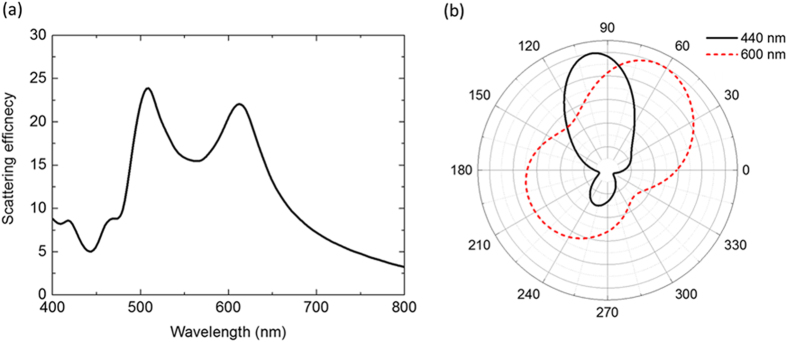
(**a**) Scattering spectrum of a dimer of silicon nanodisks on a silica substrate illuminated by an incidence propagating along the substrate with p-polarization. (**b**) Far field radiation patterns of the scattered light on the plane parallel to the substrate at λ ~ 440 nm and λ ~ 600 nm.

We performed numerical simulations of the electromagnetic behavior of the light scattered from the nanoantenna using the FDTD method. Specifically we explore a platform with a dimer of silicon nanodisks placed on a silica substrate, in line with experimentally achievable geometric dimensions. The disks have radii of 54 nm and 70 nm and a thickness of 120 nm, being separated by a distance of 20 nm. A plane wave source illuminating the structure provided electromagnetic waves propagating parallel to the substrate with the electric field polarized along the dimer axis. The scattering spectrum calculated in this configuration is shown in Fig. 6a. A small peak at λ ~ 470 nm corresponds to the electric dipolar resonance of the smaller disk. The overlap of the magnetic dipole of the smaller disk and the electric dipole of the larger disk manifests itself with a distinct peak at λ ~ 510 nm. The peak around 610 nm is associated with the magnetic dipole of the larger disk. Note that the peaks round 400 nm correspond to the magnetic quadrupolar resonances of the silicon disks. These higher order modes were neglected in the analytical dipole-dipole model used in the former section. Figure 6b shows the scattering patterns in the far field at λ = 440 nm and λ = 600 nm. While the scattered light was directed to the smaller disk at 440 nm, the scattering direction was changed in the direction of the larger disk at λ = 600 nm. Thus, the dimer of silicon nanodisks on a substrate also exhibits switchable scattering depending on the wavelength of incidence. This configuration would be quite straightforward to fabricate by top-down single-step lithography, in contrast to scattering elements based on bimetallic structures^8^,^33^.

**Figure 7:**
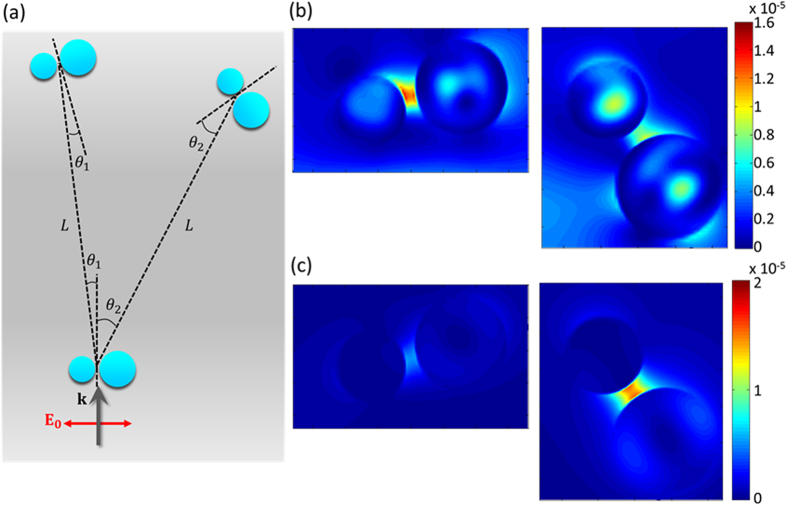
(**a**) Schematic illustration of the configuration used for the demonstration of tuneable optical guide. The first dimer was illuminated by the incident light parallel to the substrate with polarization along the dimer axis. The second dimer was placed at either left (θ_1_ = 7.2°) or right (θ_2_ = 27°) from the first one. The separation distance is L = 1.2 μm. (**b**,**c**) The electric field intensity monitored at the second dimer with the wavelength of excitation at λ ~ 440 nm (b) and λ ~ 600 nm (**c**).

This directional scattering could be used to route light propagation to selective positions, depending on the incident wavelength. To explore this possibility, we explored a configuration with two dimers using FDTD, under the same source conditions of the first dimer as for the single one (Fig. 7). The second dimer was placed at two different positions, 27° and −7° from the first dimer with a separation distance of 1.2 μm. The intensity of the electric field excited inside and around the second dimer was monitored. When the first dimer was illuminated by incident light at λ = 440 nm, the second dimer placed at −7°, showed higher electric field intensity than the other placed at 27°. In contrast, when the first dimer was illuminated by incident light at 600 nm, the second dimer placed at 27° was excited more than the other placed at −7°. This selective excitation corresponds to the far field distribution of the first dimer and its dependence on the incident wavelength. These results suggest that asymmetric dimers of high refractive index dielectrics can be used as nanoantennas which can tune the direction of the scattered field propagation by simply changing the wavelength of excitation.

In conclusion, we have demonstrated that asymmetric dimers of dielectric nanoparticles can function as nanoantennas for tuning the scattering direction of incident electromagnetic radiation by changing the incident wavelength. The excited dipoles and scattered fields have been theoretically explored using analytical dipole-dipole and phase difference models, revealing that interference between two dipoles generated perpendicular to the dimer axis is mainly responsible for the directional scattering. The dipoles perpendicular to the dimer axis can be exchanged between electric and magnetic ones depending on the polarization direction of the illuminating radiation, with interference leading to directionality in both cases. Finally, an application as a tunable nanoantenna for optical nanocircuitry has been demonstrated.

## Methods

### Theoretical calculations

The analytical dipole-dipole model^27^ was employed here to theoretically calculate the scattered field from the asymmetric dimer of silicon spherical nanoparticles. A plane wave illuminating the dimer propagates along the z-axis with electric polarization along the dimer (y) axis (p-polarized). Using this analytical dipole-dipole model, we can calculate the electric and magnetic response of the dimer but also the response of each spherical nanoparticle.


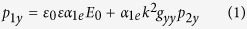



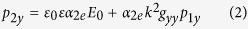



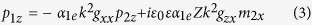



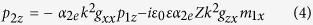



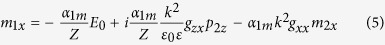



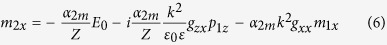


where *p*_*jy*_ and *p*_jz_ are the electric dipole moments excited in the *j*th (*j* = 1, 2) particle along the y-axis and z-axis, respectively, *m*_*jx*_ is a magnetic dipole moment along the x-axis, *ε*_0_ is the vacuum permittivity, *ε* is the relative permittivity of the loss-less media, *E*_0_ is the incident electric field, *k* is the wavenumber in vacuum, *Z* is the vacuum impedance, *α*_*je*_ and *α*_*jm*_ are the electric and magnetic polarizability of the *j*th particle, and *g*_*yy*_, *g*_*xx*_ and *g*_*zx*_ are the scalar green functions. The elements of the electric and magnetic dipoles in the two particles are given by solving these equations.

The extinction cross section can be then calculated by just considering the imaginary part of the excited dipoles in the forward direction:


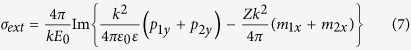


The far field distribution of the scattered field intensity per unit area is calculated from the scattered field 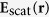 and 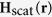 at 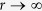,


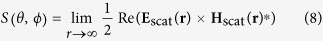


For the phase difference model, the interferences between the two magnetic dipoles are given by


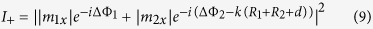



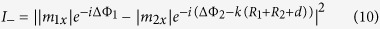


in the direction of the smaller disk (+) and the larger disk (−), respectively.

The electric and magnetic dipoles in the spherical nanoparticles excited by the *s*-polarized incidence can be calculated by the analytical dipole-dipole model and they are given by,


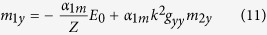



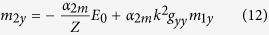



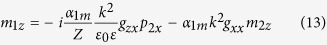



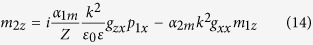



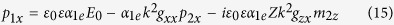



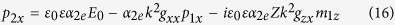


### Numerical simulations

We used a commercial FDTD solver (Lumerical Solutions) to investigate the electromagnetic behaviour of the scattered light from silicon nanodisks on a silica substrate. This numerical method is broadly established in computational electromagnetism to calculate the optical response of different nanostructures^34^. It consists of a direct implementation of the Maxwell time- dependent curl equations to solve the temporal variations of electromagnetic waves within a finite space that contains objects of arbitrary shape and properties (perfectly matched layers were used as boundary conditions). In practice, the space including the scatterer is discretized into a grid that contains the basic element of this discretization, the Yee cell. The precision of the results depends both on the number of the cells used in the simulation, as well as on the appropriate selection of the simulation time. In our case, a total-field-scattering field source was employed to remove the incident light from the scattering light. The mesh size of 1 nm and 5 nm were used in the gap of the dimer and the rest of the calculated region, respectively.

The presented results are fully converged, thus they can be considered an exact solution of Maxwell’s equations. Additionally, some of the results shown have been tested with other solving methods (FEM, using COMSOL multiphysics and the discrete dipole approximation (DDA)^35^), producing very good agreement.

## Additional Information

**How to cite this article**: Albella, P. *et al.* Switchable directional scattering of electromagnetic radiation with subwavelength asymmetric silicon dimers. *Sci. Rep.* **5**, 18322; doi: 10.1038/srep18322 (2015).

## Supplementary Material

Supplementary Information
