# Supplementary material for: Switchable directional scattering of electromagnetic radiation with subwavelength asymmetric silicon dimers
Source: Sci Rep. 2015 Dec 10;5:18322. doi: 10.1038/srep18322 (PMC4674758; doi:10.1038/srep18322)
Supplement: Supplementary Information [file srep18322-s1.doc]

**Switchable directional scattering of electromagnetic radiation with subwavelength asymmetric silicon dimers**

Pablo Albella^*,#^, Toshihiko Shibanuma* and Stefan A. Maier

The Blackett Laboratory, Imperial College London, London SW7 2AZ, UK

*These authors contributed equally to this work.

#Correspondence and requests for materials should be addressed to P. Albella

(email: p.albella@imperial.ac.uk)

**Supplementary information**

**Supplementary note 1**

**Asymmetric Silicon dimer: influence of gap size.** Extinction spectra and far field ratiation patterns calculated for a Si spherical dimer at a wavelength of 630 nm for different gap separation from 8 nm to 50, 100, 300, 600 nm (Figure S1a and S1b). As the gap was enlarged, electric dipolar resonances become narrow, which is revealed in the previous study [1]. The directionality of the scattering to the larger sphere was suppressed by increasing the gap distance, specifically the separation > 100 nm. This is because the second term of phase difference model is changed by the gap separation. Furthermore, the small gap is suitable for creating nanoantennas in terms of optical nanocircuit application.


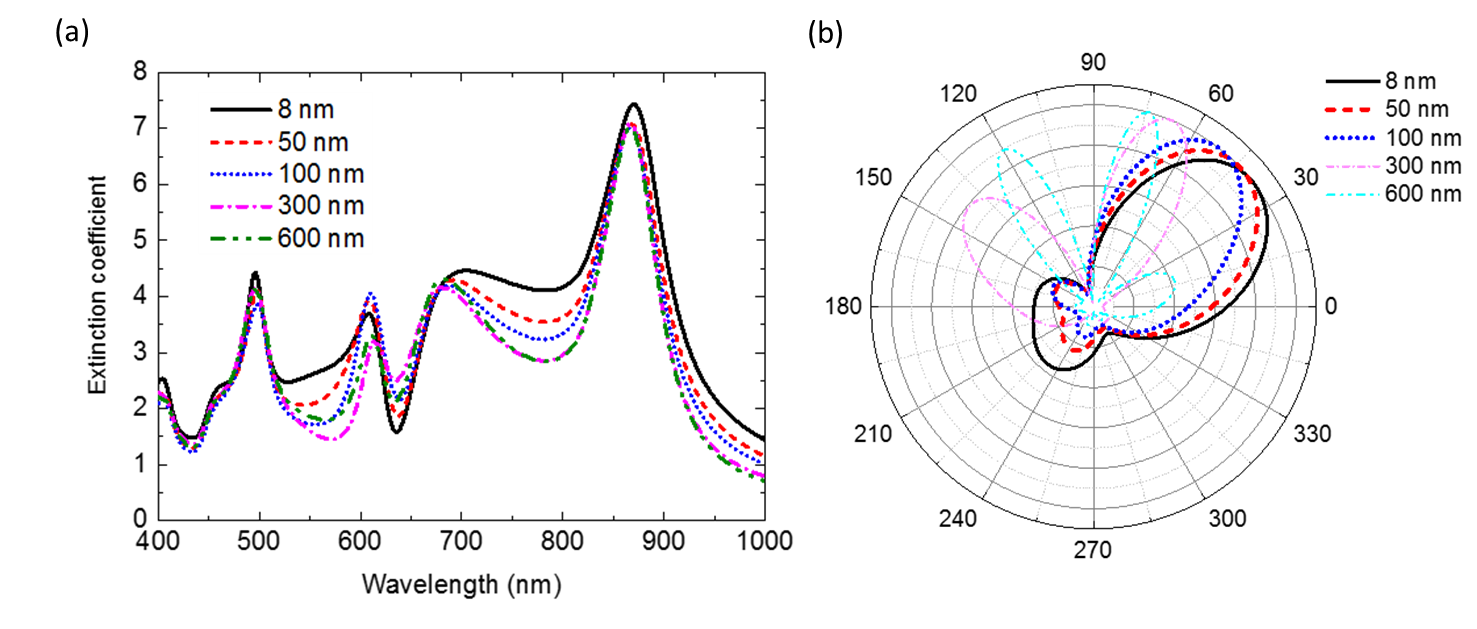


**Supplementary Figure 1 | Asymmetric Silicon dimer: influence of gap size.** (a) Extinction spectra and far field radiation patterns calculated for a Si spherical dimer at a wavelength of 630 nm for different gap separation from 8 nm to 50, 100, 300, 600 nm and (b) far field radiation patterns for the same dimer configurations.

**Supplementary note 2**

**Asymmetric Silicon dimer: influence of aspect ratio.** Figure S2a shows the extinction spectra of silicon spherical dimer where the ratio of the diameter between the two spheres, X = R1 / R2, was varied at X = 0.5 (R1 = 62.5 nm and R2 = 125 nm), 0.65 (R1 = 75 nm and R2 = 115 nm), 0.8 (R1 = 84 nm and R2 = 105 nm) and 1 (R1 = R2 = 95 nm). The position of the resonant peaks were shifted since the electric and magnetic resonant wavelengths depend on the dimension of the particle. Note that here we changed the diameter of both of the two spheres because the wavelengths at which the directional scatterings can be observed should be kept within visible regime considering the prospective application of optical nanocircuit proposed in this paper. Figure S2b shows the far field distribution of scattered fields in which the directional scattering to either left or right was achieved depending on the excitation wavelength. Since the resonant wavelengths of the magnetic dipolar modes were shifted because of the change in the geometrical dimensions, the wavelengths showing the directional scattering were also shifted. However, the steering of scattered field can be basically achieved when asymmetric silicon dimer was used, whereas the symmetric dimer (X = 1) didn’t steer the light either to left or right throughout the incident wavelengths.


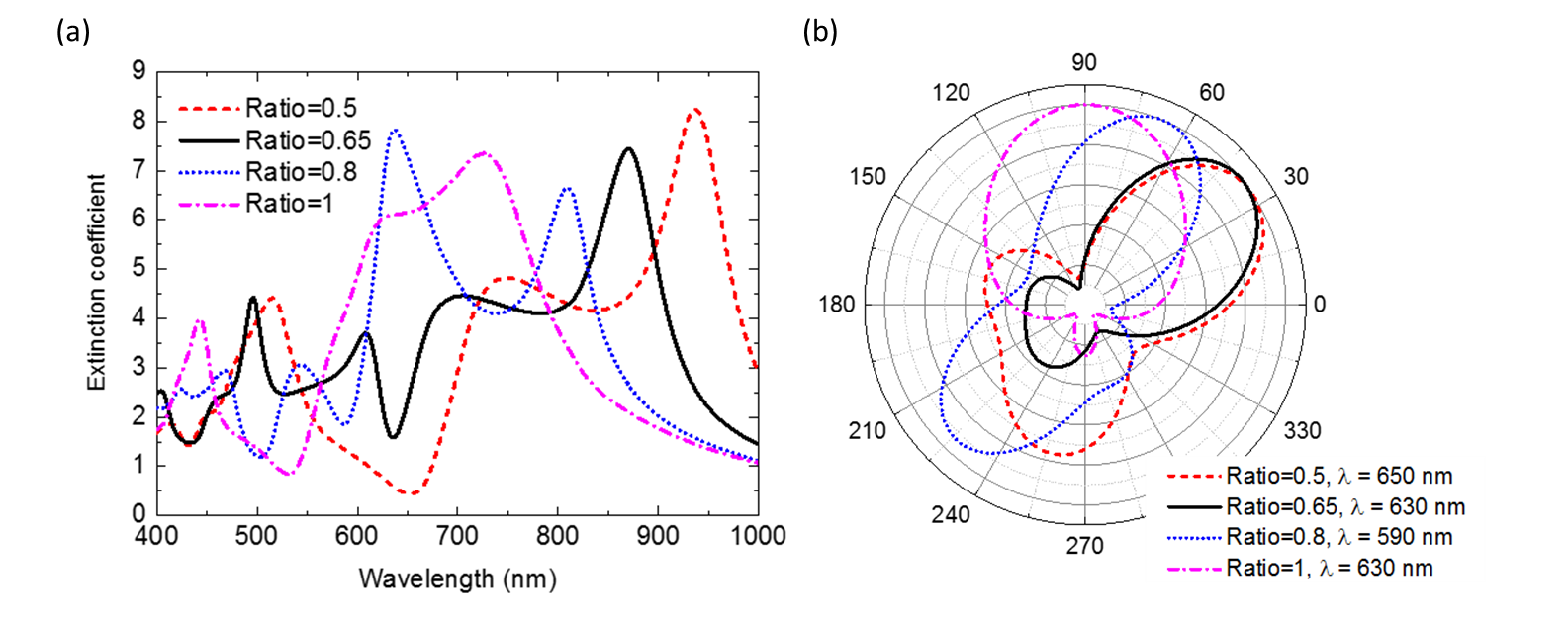


**Supplementary Figure 2 | Asymmetric Silicon dimer: influence of size ratio.** (a) Extinction spectra of silicon spherical dimer for different size ratios (X = R1 / R2). (b) Far field patterns of the scattered field at 630 nm for the same size ratios as in (a).

**Supplementary note 3**

**Comparison of the scattering direction using dipole-dipole model and FDTD method.** Figure S3 shows the angle of maximum scattering intensity in the far field radiation pattern on the y-z plane calculated by the dipole-dipole model and FDTD method. Although these two plots showed a good agreement, a new peak around 640 nm, which corresponds to the magnetic quadrupolar mode, may be causing a destructive effect for the light steering to the right direction. However, we can also see that there were also extra constructive interference in the FDTD result to enhance the degree of steering around 550 nm to the left and 850 nm to the right. A deeper study of the interaction of these modes would provide with a full understanding of the influence of higher order modes in the maximum rotation angle, however this is out of the scope of this research.


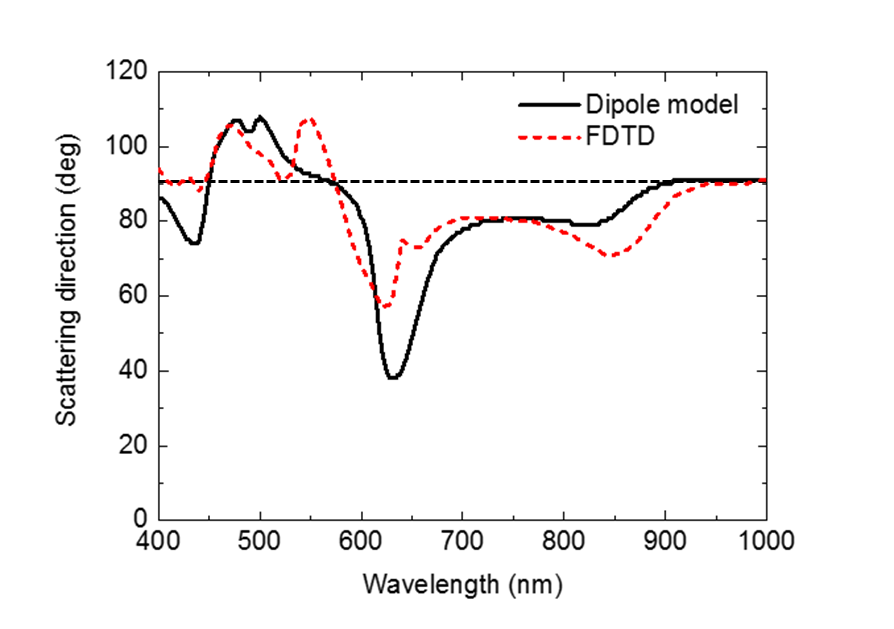


**Supplementary Figure 3 | comparison of the scattering direction using dipole-dipole model and FDTD method.** Maximum angle of the scattered filed projected on the y-z plane calculated by the dipole model (black solid line) and numerical FDTD method (red dash line).

**Supplementary References**

[1] Albella, P. et al. Low-Loss Electric and Magnetic Field-Enhanced Spectroscopy with Subwavelength Silicon Dimers. *J. Phys. Chem C* 117, 13573-13584, (2013).
